# Obituary

**Published:** 1884-06

**Authors:** 


Obituary.

The numerous friends in the Northwest of Prof. Chas. T. Hunter, A.M., M.D., late of Philadelphia, were lately pained by the report of his death, in his forty-first year. Dr. Hunter was a physician of acknowledged ability, and his early death cuts short a career of unusual brilliancy and usefulness. He was graduated from the University of Pennsylvania in 1868, taking his degree most creditably, and had since been engaged in the practice of his profession in that city. He was associated at one time with the medical Faculty of the Pennsylvania Hospital, and subsequently was elected Demonstrator of Anatomy in the University of Pennsylvania. It was while he was engaged in his professional labors that the accident occurred which has just terminated fatally, and in the loss to the profession of one of its brightest ornaments. About three years ago, while Dr. Hunter was engaged in dissection, a slight cut was inflicted upon one of his fingers, near the nail. The most skillful medical treatment failed to correct the rapidly and ominously advancing septicæmia which resulted. Recently he was removed from his city residence to his father's at Haverford College, in the hope that country air might help to allay the severity of his symptoms, but they continued to increase till the entire system was affected, and death ensued after great suffering.

Dr. Hunter's name is associated with a number of the later advances made in surgery, among which may be named the surgical use of hot water, and the revival of the transfusion of food and subcutaneous injection of fluid aliments. He was the author of the anatomical portions of Packard's and Ashbridge's works, and of numerous papers that attracted attention not only from their intrinsic merits, but also from the fact of their authorship. Among those who bore his remains to their last resting place was his old friend and physician in his last illness, Prof. D. H. Agnew.

Prof. Hunter's acquaintance was highly prized in Chicago. During his late visit to this city, a visit enforced by the condition of his failing health, he made warm friends, especially among the members of the profession to which he belonged. Many of those who met him here in the entertainments which he graced with the charm of his conversation and the ripeness of his mature intellectual powers, mourn for the loss which American medicine sustains in the death which he bravely and calmly met, joining the noble army of martyrs to the advancement of science.

